# Regulation of TGFβ Signalling by TRPV4 in Chondrocytes

**DOI:** 10.3390/cells10040726

**Published:** 2021-03-24

**Authors:** Steven Woods, Evelyn A. Humphreys, Nicola Bates, Sophie Alice Richardson, Shweta Yogesh Kuba, Imogen R. Brooks, Stuart A. Cain, Susan J. Kimber

**Affiliations:** Division of Cell Matrix Biology and Regenerative Medicine, School of Biological Sciences, Faculty of Biology, Medicine and Health, University of Manchester, Manchester M13 9PT, UK; paul.humphreys-2@postgrad.manchester.ac.uk (E.A.H.); nicola.bates@manchester.ac.uk (N.B.); sophie.richardson@manchester.ac.uk (S.A.R.); shwetayogesh.kuba@manchester.ac.uk (S.Y.K.); immobrooks@gmail.com (I.R.B.); stuart.a.cain@manchester.ac.uk (S.A.C.)

**Keywords:** TRPV4, TGFβ signalling, chondrocytes

## Abstract

The growth factor TGFβ and the mechanosensitive calcium-permeable cation channel TRPV4 are both important for the development and maintenance of many tissues. Although TRPV4 and TGFβ both affect core cellular functions, how their signals are integrated is unknown. Here we show that pharmacological activation of TRPV4 significantly increased the canonical response to TGFβ stimulation in chondrocytes. Critically, this increase was only observed when TRPV4 was activated after, but not before TGFβ stimulation. The increase was prevented by pharmacological TRPV4 inhibition or knockdown and is calcium/CamKII dependent. RNA-seq analysis after TRPV4 activation showed enrichment for the TGFβ signalling pathway and identified JUN and SP1 as key transcription factors involved in this response. TRPV4 modulation of TGFβ signalling represents an important pathway linking mechanical signalling to tissue development and homeostasis.

## 1. Introduction

TRPV4 is a calcium-permeable, non-selective cation channel [1,2] sensitive to changes in osmolality, temperature and mechanical stimulation [3,4,5]. TRPV4 is expressed in chondrocytes, where it acts as a regulator of chondrogenic differentiation [6], and continues to be active in mature chondrocytes [5], where it plays an important role in mechanical responses [7]. TRPV4 is also active during endochondral ossification [8] and plays important roles in other skeletal tissues, e.g., intervertebral disc [9]. The mechanotransduction properties of TRPV4 are essential to its regulation of the chondrocyte response to dynamic load [3]. Therefore, TRPV4 has critical functions in the formation and maintenance of healthy cartilage and this is reflected by the outcome of TRPV4 mutations, which result in pathogenic skeletal dysplasia and arthropathy [10,11]. In view of TRPV4’s apparent positive role in chondrocyte biology, it is surprising that its expression is increased in osteoarthritic (OA) cartilage [12].

The TGFβ growth factor signalling pathway also has key regulatory functions in cartilage development and homeostasis [13] and has known links to mechanical loading and the control of cell calcium [14]. TGFβ is secreted in a latent form, bound to a carrier protein, that can be sequestered in the extracellular matrix. TGFβ requires activation by liberation from the latent protein to signal by binding to type I and type II TGFβ cell-surface receptors and by forming a signalling complex that recruits and phosphorylates the transcription factors SMAD2/3. Phosphorylation of SMAD2/3 results in rapid oligomerisation with the co-SMAD (SMAD4) in a heterotrimeric complex [15]. Although SMAD proteins shuttle between the nucleus and cytoplasm even in the absence of a TGFβ signal, the formation of SMAD2/3-4 complexes drastically reduces nuclear export, which results in nuclear accumulation and causes large-scale unfolding of chromatin and increased gene transcription [16]. Active SMAD2/3-4 complexes recognise and bind to promoter regions of target genes that contain a specific 5′-AGAC-3′ SMAD-binding element (SBE) and, by association with other DNA-binding proteins, they direct specific downstream target transcription [17,18]. TGFβ can also signal through non-canonical pathways such as PI3K/AKT and MAPK signalling [19].

TGFβ signals are essential at multiple stages of chondrogenic development: for induction of mesenchymal condensation, prevention of terminal hypertrophic differentiation and maintenance of articular chondrocytes [20]. TGFβ signals work in tandem with and antagonise related Bone Morphogenetic Protein (BMP) signals to balance chondrocyte anabolic function in synthesising key matrix proteins such as type II collagen and aggrecan, or activate hypertrophic differentiation leading to cartilage calcification, matrix catabolism and vascular invasion [21,22]. It has been noted that decreased expression of TGFβ receptors is associated with ageing and in cartilage with OA [23].

TRPV4 and TGFβ thus both participate in closely related functions during cartilage development, maintenance and in disease. However, the cross talk between these pathways is unclear. In this study, we determined how these pathways interact and illuminate some of the molecular mechanisms involved.

## 2. Materials and Methods

### 2.1. Chondrocyte Isolation and Culture

Human TC28a2 chondrocytes [24], bovine articular chondrocytes and the mouse ATDC5 chondrogenic cell line were maintained in Dulbecco’s Modified Eagle Medium (DMEM) (Gibco, Thermo Fisher Scientific, Altrincham, UK) containing 10% heat-inactivated Foetal Bovine Serum (Sigma-Aldrich, Cambridge, UK) and 2 mM L-glutamine (Sigma-Aldrich) at 37 °C, 5% CO_2_, unless otherwise stated.

Bovine articular chondrocytes were isolated from stifle joints as previously described [25]. Briefly, articular cartilage was cut into 2–3 mm pieces, enzymatically digested in serum-free medium containing 0.5% pronase for one hour and then transferred to serum-free medium containing 0.5% collagenase type II and 0.1% hyaluronidase for two to three hours on an orbital shaker at 37 °C. Supernatant was then passed through a 40 µm filter and centrifuged at 500 g for 5 min to collect the isolated bovine articular chondrocytes.

### 2.2. Cell Stimulation

Prior to stimulation, cells were serum starved overnight (~16 h) using DMEM + 2 mM L-glutamine (without serum). Unless otherwise stated, cells were stimulated with 10 ng/mL TGFβ1 (Cat#100-21C, PeproTech, London, UK) or TGFβ3 (Cat#100-36E, PeproTech) for TGFβ pathway stimulation and then after 15 min with 100 nM GSK101 (GSK1016790A, Sigma-Aldrich) to activate TRPV4. For TRPV4 inhibition, GSK219 (GSK2193874, Sigma-Aldrich) was added along with TGFβ3 (15 min before GSK101). For reduced calcium experiments, DMEM with calcium (1.8 mM) was serial diluted 5 fold with calcium-free DMEM, corresponding to calcium concentrations of 360, 72, 14.4 and 2.8 µM calcium. The switch to reduced calcium (no serum) was performed ~16 h prior to stimulation. For calmodulin inhibition experiments, KN93 was also added to cells during serum starvation (~16 h prior to stimulation). For experiments involving GSK101, GSK219 or KN93, control cells were treated with the equivalent amount of DMSO to act as a vehicle control.

### 2.3. Immunofluorescence

TC28a2 were seeded at a density of 30,000 cells per well in a 24-well plate, grown for 24 h and then serum starved overnight. Cells were washed twice with PBS, then fixed with 4% paraformaldehyde (PFA) for 15 min at room temperature. Cells were washed a further three times with PBS. All wells were blocked and permeabilised using 10% donkey serum and 0.1% Triton X-100 (Sigma-Aldrich) in a solution of 0.05% TWEEN-20 (Sigma-Aldrich), 0.1% BSA in PBS for 30 min. Primary antibodies were diluted in a solution of 0.05% TWEEN-20, 0.1% BSA and PBS with 1% donkey serum and 0.1% Triton X. Cells were then incubated with TRPV4 antibody (1:200, Cat#ACC-124, Alomone Labs, Jerusalem, Israel) or Rabbit IgG control (1:200, Cell Signalling Technologies, London, UK) overnight. Cells were washed three times with a solution of 0.05% TWEEN-20, 0.1% BSA in PBS prior to the addition of the secondary antibody (1:200, Invitrogen, Thermo Fisher ScientifiC, Altrincham, UK), which was diluted in a solution of 0.05% TWEEN-20, 0.1% BSA in PBS with 1% donkey serum and 0.1% Triton X-100. A further three washes were performed with a solution of 0.05% TWEEN-20, 0.1% BSA in PBS, then cells were stained with 4″6′Diamidine-2′-phenylindole dichloride (DAPI, Invitrogen, 1:500) diluted in PBS for 5 min. A final three washes with PBS were undertaken prior to imaging using an inverted BX51 microscope (Olympus Southend-on-Sea, UK) equipped with a Q-Imaging camera (Micro Imaging Applications Group Inc., Surrey, British Columbia, Canada). Images were captured with the Q-Capture Pro software package version 6 (Micro Imaging Applications Group Inc.).

### 2.4. Calcium Imaging and Quantification

TC28a2 cells were cultured overnight at a density of 30,000 cells per well in a 24-well plate for image analysis or 10,000 cells per well in a 96-well plate for quantification using GloMax^®^-Multi+ Detection System (Promega, Madison, WI, USA). Cells were then serum starved overnight. Fluo8AM (Invitrogen) was diluted to 5 mM in DMSO. The Fluo 8AM was then diluted to 5 µM in DMEM before addition to cells. Cells were incubated in 5 µM Fluo8 for 30 min before washing with DMEM three times. Cells were incubated for a further 30 min prior to fluorescent imaging with the Leica DFC365 FX microscope with the Leica Application Suite (Leica Wetzlar, Germany) or fluorescence detection on the GloMax^®^-Multi+ Detection System (Promega).

### 2.5. Generation of SBE Reporter TC28a2 Cell Line

A lentiviral TGFβ signalling reporter vector was constructed using the SMAD-binding element (SBE) based on the 8 bp palindromic sequence recognised by SMAD3 and SMAD4, in bold below [26]. The reporter promoter, which consists of four AGTAT**GTCTAGAC**TG DNA repeats, was placed upstream of a minimal reporter element. The SBE promoter was cloned upstream of a destabilised form of NanoLuc Luciferase (Promega) containing a C-terminal protein degradation sequence (PEST sequence, NLucP). To generate red fluorescence protein-positive (RFP+) cells with NLucP driven by SBE, the SBE-NLucP was first cloned into the lentiviral expression vector pCDH-EF1α-tagRFP (SBI Systems Bioscience, CA, USA), resulting in the construct pCDH-SBE-nLUCP-EF1a-tagRFP (Figure S1). Lentiviral particles were generated in HEK293T cells. TC28a2 cells were fluorescence-activated cell sorted (FACS) for RFP-positive cells following their transduction, using BD FACsAria^TM^ Fusion cell sorter.

### 2.6. Luciferase Reporter Assay

A total of 10,000 cells were seeded in each well of a black-walled 96-well plate and then serum starved and stimulated as described above. Nano-Luc luciferase expression was detected using the Nanoglo Live reagent and GloMax^®^-Multi+ Detection System (Promega).

### 2.7. Western Blotting

TC28a2 cells were plated at 150,000 cells per well of a 6-well plate in DMEM containing 10% serum medium (as stated above) and cultured for 48 h before changing to serum-free medium for 16 h. Time–course experiments were conducted using either GSK101 activator (100 nM) or TGFβ3 (PeproTech EC 10 ng/mL) for 4 h, 2 h, 1 h, 30 min, 15 min, or no addition control. Samples were washed briefly with PBS, and protein extracted at the designated time Cell Lysis buffer with 6µM PMSF (CST 9803 and CST 8553) added prior to use. Plates were incubated for 5 min on ice, before scraping into Eppendorf tubes. Samples were centrifuged at 14,000× *g* 10 min at 4 °C and supernatant was stored at −20 °C until required.

Protein concentration was measured using the Pierce BCA Assay kit (Pierce, Thermo Fisher Scientific, Altrincham, UK) and 20 µg samples were boiled with Pierce lane marker-reducing buffer (Thermo 39,000) at 95 °C for 10 min. Samples were run on 10% Bis-Tris gels (Thermo NW00100BOX) with broad range markers (11–245 KDa, NEB P7712S). Protein was transferred using the iBlot-2 Gel Transfer device (Thermo IB21001), using iBlot-2 Transfer stacks (nitrocellulose membrane, Thermo IB23001). Membranes were blocked for 1 h at room temperature with 5% BSA in 1x TBS-0.1%Tween-20, then probed with antibodies for SMAD2 (1:1000, CST 5339), pSMAD2 (1:1000, CST 18338), TRPV4 (1:200, Cat#ACC-124, Alomone Labs) and left overnight on a rocker at 4 °C. Membranes were washed three times with TBS-0.1%Tween-20, then secondary antibodies were added (1:15,000 IRDye 800CW Donkey anti-Rabbit; LI-COR 926-32213) in 5% BSA in TBS-0.1%Tween-20. After three washes in TBS-0.1%Tween-20, staining was imaged and analysed using the Odyssey CLx imaging system (LI-COR) and ImageStudio Lite software. GAPDH (1:1000 CST 5174S) was used as a loading control. TRPV4 blocking peptide (#BLP-CC124) was reconstituted as per manufacturer’s instructions with double-distilled water. Parallel membranes were incubated with either only antibody for TRPV4 or antibody for TRPV4 plus blocking peptide (according to manufacturer’s instructions. Membranes were then incubated at 4 °C overnight on a rocker, followed by Western blotting as stated above.

### 2.8. SiRNA Transfection

TC28a2 cells were plated at a density of 10,000 per well of a 96-well plate and incubated for 3 h, after which time cells had adhered. Cells were then transfected using RNAiMAX and siRNA for 24 h (final mix containing 10 nM siRNA and 10% FBS). Transfection mix was then removed and cells were serum starved for 2 days prior to stimulation.

### 2.9. RNA-Seq

For RNA-seq experiments, TC28a2 chondrocytes were plated onto 6-well plates at a density of 150,000 cells per well and grown in DMEM+10%FCS for 24 h, then serum starved overnight. Cells were then stimulated with 10 ng/mL TGFβ3, and/or 100 nM GSK101 for TRPV4 activation, and/or 500 nM GSK219 for TRPV4 inhibition. GSK101 was added 15 min after TGFβ as we found that GSK101 enhanced TGFβ signalling at this time. GSK219 was added along with TGFβ3 (15 min before GSK101), as when GSK219 was added at later timepoints (along with GSK101) it was ineffective against GSK101. Cells were lysed and total RNA isolated using the mirVana isolation kit (Thermofisher) 4 h post TGFβ stimulation (3 h 45 m post GSK101). Three independent experiments were performed and RNA isolated from each. RNA-seq was performed using Illumina HiSeq4000. Unmapped paired-reads of 76 bp were interrogated using a quality control pipeline consisting of FastQC v0.11.3 (http://www.bioinformatics.babraham.ac.uk/projects/fastqc/ (accessed on 7 October 2017) and FastQ Screen v0.9.2 (http://www.bioinformatics.babraham.ac.uk/projects/fastq_screen/ (accessed on 7 October 2017). The reads were trimmed to remove any adapter or poor quality sequence using Trimmomatic v0.36 [27]; reads were truncated at a sliding 4 bp window, starting at 5′, with a mean quality < Q20, and removed if the final length was less than 35 bp. The filtered reads were mapped to the human reference sequence analysis set (hg38/December 2013/GRCh38) from the UCSC browser, using STAR v2.7.2b [28]. The genome index was created using the comprehensive Gencode v32 gene annotation (http://www.gencodegenes.org/ (accessed on 18 September 2019) [29]. The flag ‘quantMode GeneCounts’ was used to generate read counts into genes. Normalisation and differential expression analysis was performed using DESeq2 v1.10.1 on R v3.2.3, Normalisation was performed using a median of ratios method and p values were calculated using the Wald test and adjusted for multiple testing using the procedure of Benjamini–Hochberg [30].

### 2.10. Pathway Analysis

Genes whose expression significantly increased following either TRPV4 activation (1448 genes), TGFβ stimulation (1991 genes) or TRPV4 activation of cells previously stimulated with TGFβ (1812 genes) were analysed for enrichment of Kegg pathways, Wiki pathways and ChEA transcription factors using EnrichR [31], and p values were adjusted for multiple testing using the procedure of Benjamini–Hochberg.

### 2.11. Reverse Transcription and qRT-PCR

RNA extraction for qRT-PCR was performed using Qiagen RNeasy mini-kit following manufacturer’s instructions (Qiagen 74,104). Reverse transcription was performed using the High-Capacity cDNA Reverse Transcription Kit (Thermo Fisher Scientific 4368813). qPCR reaction was performed using PowerUp SYBR green master mix (ThermoFisher Scientific, #A25742) and gene-specific primers (Table S1) with the following cycling conditions on a BioRad C1000Touch Thermal Cycler: denaturation at 95 °C for 10 min, 39 cycles of 95 °C for 30 s, 60 °C for 30 s and 72 °C for 35 s, final extension at 72 °C for 10 min, and melt curve analysis at 65 °C for 5 s and 95 °C for 30 s. Gene expression was normalised to GAPDH using the 2−ΔCT method.

### 2.12. Transcription Factor-Binding Motif Analysis

Identification of transcription factor-binding sites present in significantly upregulated genes and annotation of corresponding motif sequences (TRANSFAC database) were carried out with g:Profiler [32]. Motif sequences of corresponding transcription factors were further compared with in silico predicted and experimentally validated DNA-binding motifs collection databases (JASPAR2018_CORE_vertebrates_non-redundant, Jolma 2013, TRANSFAC, UniPROBE mouse, HOCOMOCO v11 and Swiss Regulon) using TOMTOM (e-value 0.5; min overlap between motifs) [33].

## 3. Results

### 3.1. Expression and Activation of TRPV4 in TC28a2 Chondrocytes

**Figure 1:**
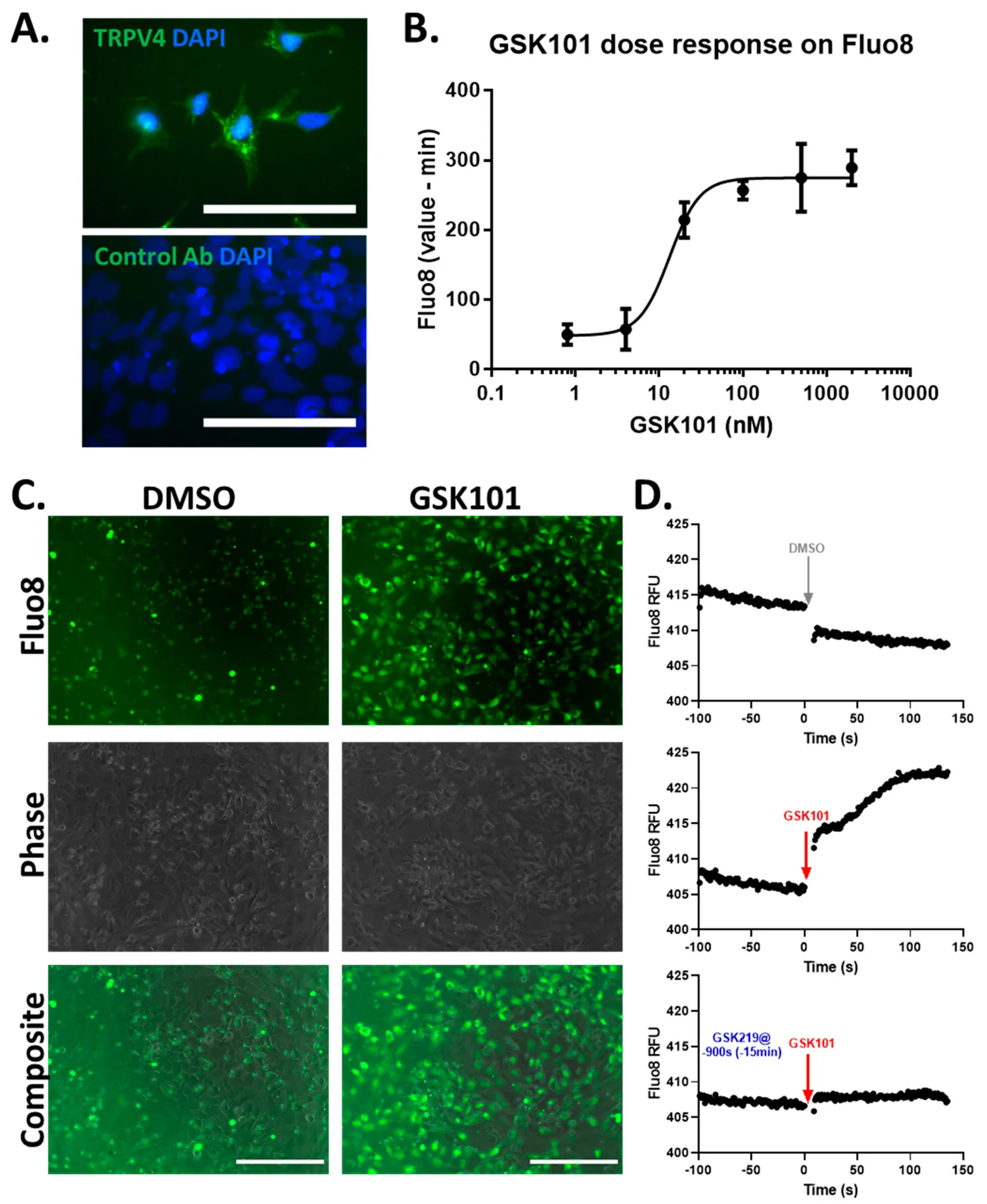
TRPV4 is expressed and can be activated in TC28a2 chondrocytes. (**A**) Immunostaining for TRPV4 in TC28a2 chondrocytes, using anti-TRPV4 antibody and DAPI nuclear stain. Scale bar represents 100 µm. (**B**) Dose response of GSK101 on Fluo8 fluorescence 15 min post stimulation. (**C**) Fluorescence imaging of Fluo8-loaded TC28a2 cells 15 min post stimulation with 100 nM GSK101 or DMSO control. Scale bar represents 200 µm. (**D**) Representative traces of Fluo8 fluorescence following DMSO (upper), 100 nM GSK101 stimulation (middle) or 100 nM GSK101 stimulation in cells pre-incubated with 500 nM GSK219 (lower) for 15 min. Data representative of three independent experiments.

To investigate how TRPV4 activity affects TGFβ signalling, we first chose to use a human chondrocyte cell line, TC28a2, which expresses TRPV4 and is known to respond to TGFβ. TC28a2 cells are immortalised and have a more stable pattern of gene expression in contrast to primary chondrocytes, which show age-related changes in proliferation and gene expression as they de-differentiate when cultured [34]. As evidence for their suitability, TC28a2 cells were tested for TRPV4 expression using Western blotting (Figure S2) and immunofluorescence, showing all TC28a2 cells expressed some TRPV4 protein (Figure 1A). To demonstrate TRPV4 activation TC28a2 cells were loaded with the fluorescent Fluo8 calcium indicator and stimulated with a selective TRPV4 activator GSK101 [35]. This rapidly increased intracellular calcium (<1 min), with an EC50 of 13.3 ± 2.9 nM (Figure 1B,C), an increase which was completely prevented by pre-incubation of cells with the selective TRPV4 inhibitor GSK219 [36] (Figure 1D).

### 3.2. Validation of SBE Reporter in TC28a2 Chondrocytes

To investigate the interaction between TRPV4 and TGFβ signalling, we created a TC28a2 cell line with a TGFβ signalling reporter (SBE-nLUCp see methods). Induction of SBE-nLUCp activity in TC28a2 cells was associated with both a dose and temporal response to TGFβ3 (Figure S1B,C), which was validated by pSMAD2 immunoblotting. As expected, phosphorylation of SMAD2 peaked slightly earlier than SBE-nLUCp activity (Figure S1D). SBE-nLUCp activity was consistently detected 4 h post stimulation with 10 ng/mL TGFβ3 (Figure S1C), and these conditions were therefore used routinely to determine TGFβ pathway activity.

### 3.3. Activation of TRPV4 Enhances TGFβ Signalling

**Figure 2:**
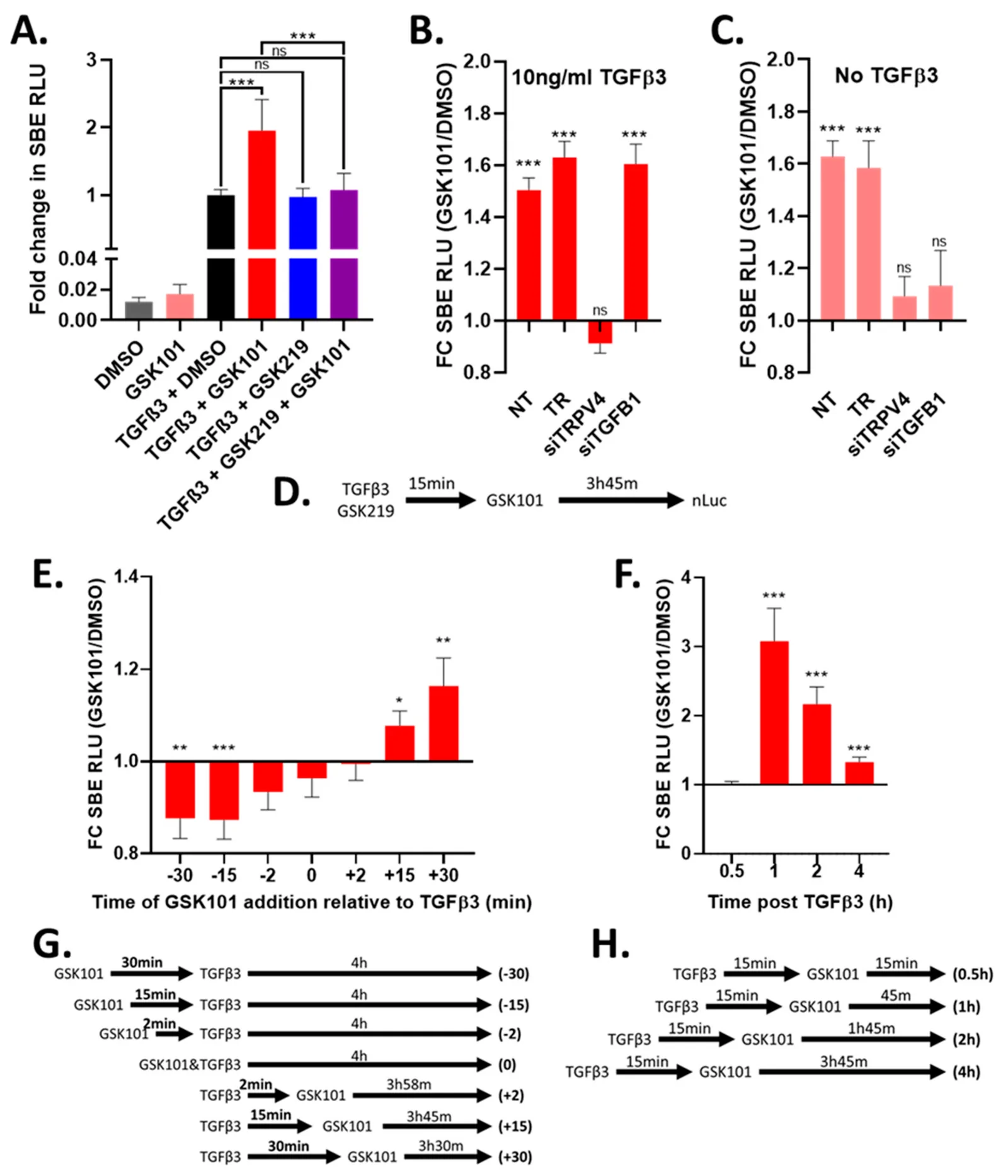
Activation of TRPV4 modulates TGFβ signalling in a time-dependent manor. TC28a2 cells with SBE-nLUCp reporter were used to monitor TGFβ signalling. (**A**) Cells were stimulated with 10 ng/mL TGFβ3 or medium control, incubated for 15 min then stimulated with 100 nM GSK101 (activator) or DMSO control (vehicle) and then incubated for a further 3 h 45 min before SBE-nLUCp activity was determined. TRPV4 inhibitor (500 nM GSK219) was added to cells along with TGFβ3. (**B**) and (**C**) Cells were either not transfected (NT), mock transfected (TR), transfected with siRNA to TGFB1 (siTGFB1) or transfected with siRNA to TRPV4 (siTRPV4) for 24 h and then serum starved and incubated for a further 48 h. Following incubation, cells were stimulated with TGFβ3 (**B**) or media control (**C**) and then TRPV4 activated using GSK101, SBE-nLUCp activity determined as described in (**A**). (**D**) Schematic illustrating the order of stimulation/activation for A–C. (**E**) Cells were stimulated with 10 ng/mL TGFβ3. TRPV4 was activated (100 nM GSK101/DMSO control) either before (-ve mins), with (0 min) or after (+ve mins) TGFβ3 stimulation. (**F**) Cells were stimulated with 10 ng/mL TGFβ3 or medium control, incubated for 15 min then TRPV4 activated using 100 nM GSK101 or DMSO control. SBE-nLUCp activity was determined after the indicated amount of time post TGFβ3 stimulation. (**G**) Schematic representation of conditions shown in E. (**H**) Schematic representation of conditions shown in F. FC SBE RLU; fold change in SMAD-binding element relative light units NT; no treatment. Data in A combined from four independent experiments, and data in B–F combined from three independent experiments. Raw data are shown in Figures S3 and S5. GSK101 treatment was normalised to the DMSO control for each siRNA/timepoint. Statistical differences were calculated by two-way ANOVA followed by Sidak’s multiple comparisons test; *p* < 0.05 *, *p* < 0.01 **, and *p* < 0.001 ***.

TRPV4 can be activated by selective agonists such as GSK101 and when TGFβ reporter cells were tested with GSK101 following TGFβ3 stimulation, it was found to enhance TGFβ signalling, as shown by increased SBE-nLUCp activity (Figure 2A and Figure S3A) and increased nuclear translocation of SMAD2 (Figure S4A,B). This GSK101 effect was saturated at 100 nM (Figure S3A) and this concentration was used for subsequent experiments. GSK219 did not affect TGFβ3-induced SBE-nLUCp activity (Figure 2A and Figure S3B), but it was able to block the GSK101 increase in TGFβ3-induced SBE-nLUCp activity (Figure 2A and Figure S3C). Based on these data, 500 nM GSK219 was used in subsequent experiments to ensure effective TRPV4 inhibition. Using siRNA to TRPV4 prevented the GSK101 enhancement of TGFβ signalling, showing that the effect was indeed facilitated entirely through TRPV4 (Figure 2B and Figure S3D).

TGFβ signalling showed a small increase when TRPV4 was activated, even in the absence of added TGFβ3 (Figure 2A). This was likely due to low endogenous expression of TGFβ1 as it could be prevented by siRNAs to TRPV4 or TGFB1 (Figure 2C and Figure S3E). Importantly siRNA to TGFB1 did not prevent TRPV4-mediated enhancement of TGFβ signalling in the presence of exogenous TGFβ3 (Figure 2B). Furthermore, TRPV4 activation also enhanced TGFβ signalling mediated by exogenous TGFβ1, showing that the effect of TRPV4 was unaffected by which TGFβ ligand was driving the TGFβ signalling (Figure S4C,D). Using the same TGFβ reporter in primary bovine articular chondrocytes and murine ATDC5 cells we showed TRPV4 activation following TGFβ3 stimulation enhanced SBE-nLUCp activity, demonstrating that TRPV4 modulation of TGFβ signalling in chondrocytes is conserved across species (Figure S4E,F).

### 3.4. Timing of TRPV4 Activation and Inhibition Is Important for TGFβ Signalling Activity

As it was unclear at what part of the TGFβ signalling cascade TRPV4 might be having its effect, we investigated the sequence and timing of responses. In our data reported above, when TRPV4 was activated 15 min after TGFβ3 stimulation, an enhancement of TGFβ signalling activity was observed (Figure 2A,B). To determine over what time scale TRPV4 activation affected TGFβ signalling, we activated TRPV4 at different times relative to TGFβ3 stimulation and then measured SBE-nLUCp activity 4 h post TGFβ3 stimulation. Consistent with Figure 2A, activation of TRPV4 15 or 30 min after TGFβ3 stimulation enhanced TGFβ signalling (Figure 2E). However, TRPV4 activation 15 or 30 min prior to TGFβ3 stimulation had the opposite effect and significantly reduced TGFβ signalling (Figure 2E and Figure S5). The addition of TRPV4 activator close to TGFβ3 (±2 min), had no significant effect (Figure 2E). These data demonstrate that the effect of TRPV4 is rapid, transient and dependent upon the stage of TGFβ signal transduction.

When TRPV4 was activated after TGFβ3 stimulation, an increase in TGFβ signalling was consistently observed 4 h post stimulation (Figure 2). Consistent with the observed rapid nuclear SMAD2 translocation (Figure S4), SBE activity showed enhancement as early as 1 h post TGFβ stimulation (45 min post TRPV4 activation), which then decreases with time (Figure 2F), and this decrease was also observed for primary bovine articular chondrocytes and murine ATDC5 cells (Figure S4). Most likely, this was about the earliest timepoint at which TGFβ3 can activate a transcriptional response since no enhanced SBE response was seen at 30 min (Figure 2F). These data indicate that the mechanism by which TRPV4 activation enhances TGFβ signalling results in changes in gene expression output within 45 min, but it has no effect, or a negative effect if TRPV4 activation occurs before the TGFβ signalling cascade is active.

### 3.5. RNA-Seq Identification of the TGFβ3 Response Genes Enhanced by TRPV4 Activation

**Figure 3:**
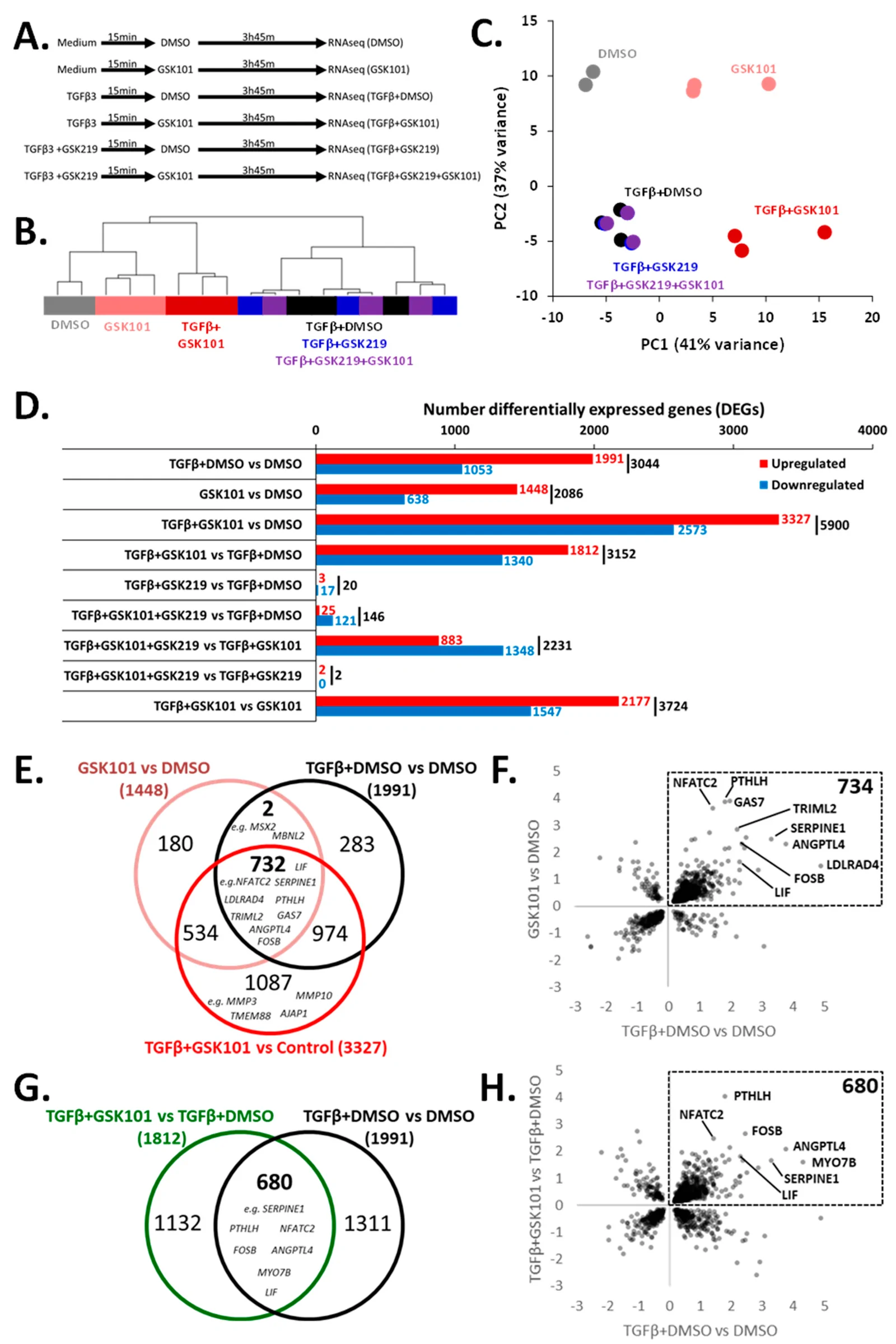
RNA-seq identification of TGFβ3 response genes that are enhanced by TRPV4 activation. (**A**) Experimental design for RNA-seq (triplicate). (**B**) Hierarchical clustering and (**C**) PCA analysis shows separation of DMSO, GSK101, TGFβ3+DMSO and TGFβ3+GSK101 treatment groups, the TGFβ3+GSK219 and TGFβ3+GSK219+GSK101 treatment groups both clustered with TGFβ3+DMSO. (**D**) Histogram indicating number of differentially expressed genes (DEGs) between experimental conditions according to DESeq2. (**E**) Venn diagram indicating commonality between genes significantly up regulated in GSK101 vs. DMSO, TGFβ3+DMSO vs. DMSO and TGFβ3+GSK101 vs. DMSO. (**F**) Scatter plot of significant genes comparing fold change in gene expression in GSK101 vs. DMSO and TGFβ3+DMSO vs. DMSO. (**G**) Venn diagram of genes significantly up regulated in TGFβ3+GSK101 vs. TGFβ3+DMSO or TGFβ3+DMSO vs. DMSO illustrating that GSK101 causes further enhancement of TGFβ response genes. (**H**) Scatter plot of significant genes comparing fold change in gene expression following TGFβ3+DMSO vs. DMSO and TGFβ3+GSK101 vs. TGFβ3+DMSO.

To determine the effect of TRPV4 activation and establish its impact on TGFβ signalling, we performed RNA-seq on TC28a2 cells following TRPV4 activation/inhibition in the presence and absence of prior TGFβ3 stimulation (Figure 3A). Hierarchical clustering and PCA analysis showed clear separation of control (DMSO), GSK101, TGFβ3+DMSO and TGFβ3+GSK101 treatment groups (Figure 3B,C). The TGFβ3+GSK219 and TGFβ3+GSK219+GSK101 treatment groups both clustered with TGFβ3+DMSO, confirming GSK219 inhibition of TRPV4 activation (Figure 3B,C).

Differential gene expression analysis was performed across experimental conditions (Figure 3D, Table S2). Comparison of TGFβ3+DMSO vs. DMSO, GSK101 vs. DMSO, TGFβ3+GSK101 vs. DMSO and TGFβ3+GSK101 vs. TGFβ3+DMSO revealed 1991, 1448, 3327 and 1812 genes, respectively, whose expression significantly increased (Table S3). Although the majority of the 3327 genes increased by TGFβ+GSK101 were increased with either the addition of TGFβ3 or GSK101 individually, there were 1087 genes significantly increased by TGFβ3+GSK101, that were not significantly increased by either TGFβ3 or GSK101 individually. These included *MMP3*, *MMP10*, *TMEM88* and *AJAP1* (Figure 3E and Figure S6A). There was also a notable overlap between the genes significantly changed after TGFβ3 and GSK101, with 734 genes significantly up regulated in both TGFβ3 stimulation and TRPV4 activation (Figure 3E,F). Furthermore, because we demonstrated an enhancement of SBE-nLUCp induction by TRPV4 activation after TGFβ3 stimulation, we hypothesised that there would be many genes whose expression was increased by TGFβ3 that would be further enhanced by TRPV4 activation. Indeed, of the 1991 genes increased in response to TGFβ3, 680 were further enhanced by TRPV4 activation (including *LIF*, *SERPINE1*, *ANGPTL4*, *PTHLH*, *NFATC2* and *GAS7*; Figure 3G,H). Stimulation with both TGFβ3 and GSK101 induced a response which was greater than the sum of the responses to individual factors for some genes (e.g., *LIF*, *SERPINE1*, *ANGPTL4* and *PTHLH*), suggesting a synergistic relationship between TGFβ3 and TRPV4 activation (Figure S6B). Others (e.g., *NFATC2* and *GAS7*) were predominantly increased by TRPV4 activation (Figure S6C). These data indicate that TRPV4 activation can increase expression of some TGFβ3 response genes.

Other gene expression patterns included genes that were predominantly sensitive to TRPV4 activation but not to TGFβ3 stimulation such as *BMPER* and *TMEM158* (Figure S6C) and conversely genes that were responsive to TGFβ3 stimulation but not to TRPV4 activation included *NEDD9*, *GAL*, *KANK4* and *LDLRAD4* (Figure S6D). There was also a group of genes where TGFβ3 stimulation and TRPV4 activation decreased expression such as *TXNIP*, *BMP4*, *TGFBR3* and *GDF5* (Figure S6E). Changes in gene expression of example genes were validated using q-RT-PCR (Figure S7). Comparison of TGFβ3+GSK219 vs. TGFβ3+DMSO and TGFβ3+GSK101+GSK219 vs. TGFβ3+DMSO revealed only 20 and 146 DEGs, respectively (Figure 3D, Table S2), suggesting that the TRPV4 inhibitor GSK219 has very little effect on the TGFβ pathway, yet can almost completely suppress the effect of TRPV4 activation on TGFβ3 signalling.

### 3.6. Pathway Analysis

To elucidate the downstream consequences of TGFβ3 stimulation and TRPV4 activation, we performed pathway analysis on genes significantly upregulated in the following comparisons: TGFβ3+DMSO vs. DMSO (1991 genes), GSK101 vs. DMSO (1448 genes) and TGFβ3+GSK101 vs. TGFβ3+DMSO (1812 genes) (Tables S3 and S4). As expected, ‘TGF-beta signalling pathway’ was significantly enriched in genes upregulated following TGFβ3 stimulation (KEGG padj = 1.10 × 10^−5^, Table S4, Figure S8A). The pathway analysis demonstrated that ‘TGF-beta signalling pathway’ was also significantly enriched following TRPV4 activation, even in the absence of exogenous TGFβ (KEGG padj = 0.0022790, Table S4, Figure S8B). As noted above, many of the Kegg ‘TGF-beta signalling pathway’ genes were upregulated by both TGFβ3 stimulation and TRPV4 activation (e.g., *TGFB2*, *INHBA*, *LTBP1* and *MYC*), although some genes in the pathway (e.g., *SMAD7*, *BMPR2* and *CDKN2B*) are only upregulated by TGFβ3, while others (e.g., *FST* and *NOG*) are up regulated by TRPV4 activation to a greater extent than by TGFβ stimulation (Table S4, Figure S9). *BAMBI*, an inhibitor of the TGFβ pathway was also downregulated by TRPV4 activation. Other enriched pathways involving genes upregulated following TRPV4 activation include ‘focal adhesion’ (padj = 3.64 × 10^−10^; Table S4, Figure S10), which is important for TGFβ regulation [37], as well as the ‘PI3K/Akt signalling pathway’ (padj = 2.84 × 10^−7^; Table S4, Figure S11) and the ‘MAPK signalling pathway’ (padj = 0.00058; Table S4, Figure S12). These appear consistent with previous reports showing TRPV4 activation of non-canonical TGFβ signalling pathways [38,39].

### 3.7. TRPV4 Activation Enhances TGFβ Signalling through Calcium/Calmodulin

**Figure 4:**
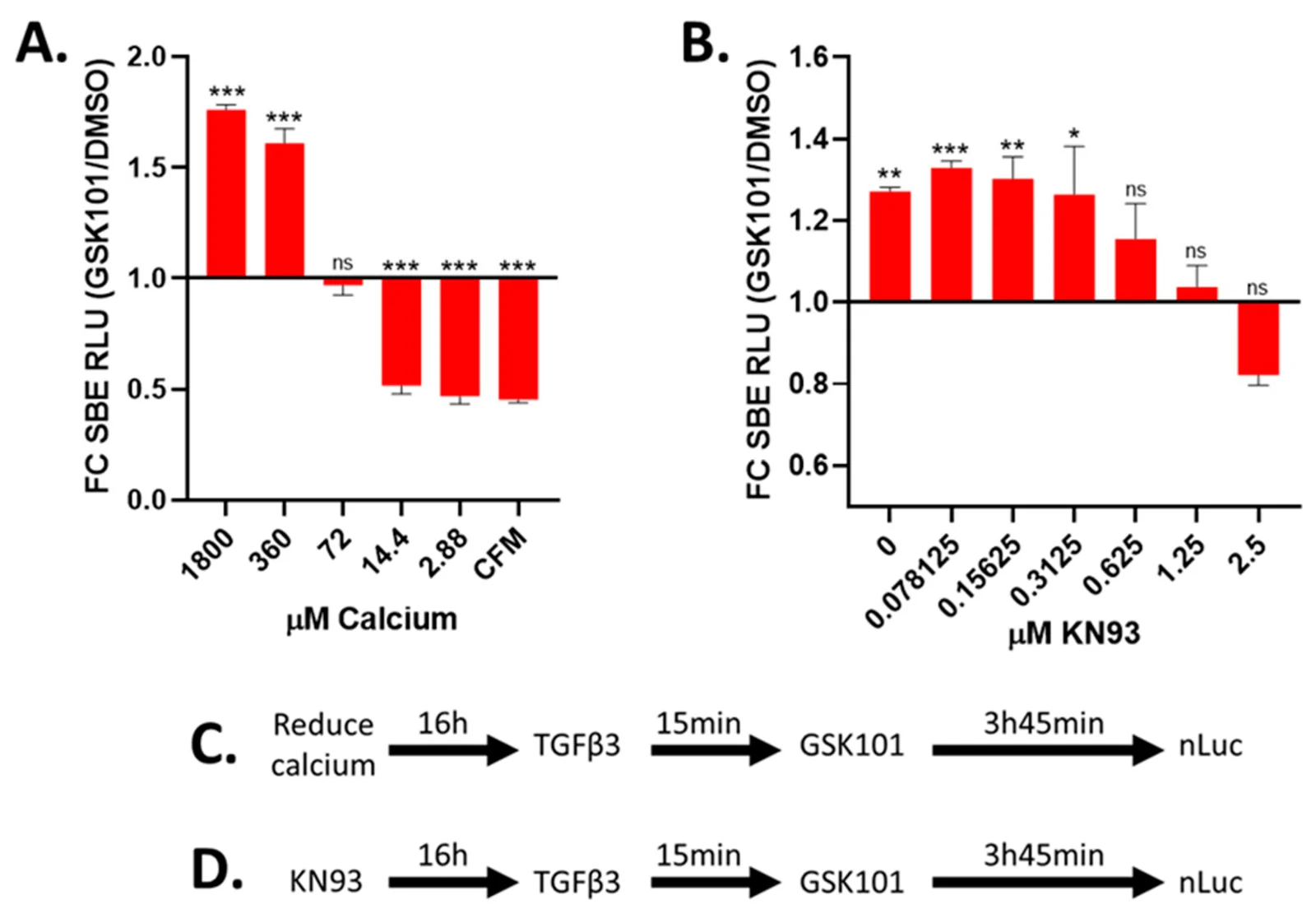
Reduction in extracellular calcium or calmodulin inhibition prevents GSK101 enhancement of TGFβ signalling. TC28a2 cells grown with indicated concentration of calcium (**A**) or KN93 (**B**) for ~16 h and then stimulated with TGFβ3 followed by DMSO (black) or GSK101 (red) after 15 min, and luciferase activity was determined 4 h after TGFβ3. (**A**) TRPV4 activation (using 100 nM GSK101) does not enhance TGFβ signalling at low calcium concentrations in medium. (**B**) Pre-treatment with calmodulin inhibitor (KN93) prevents TRPV4 activation (using 100 nM GSK101) of enhanced TGFβ signalling. (**C**,**D**) Schematics showing timing for calcium removal or calmodulin inhibition (KN93) in relation to stimulation/activation. Data were combined from three independent experiments. CFM; calcium-free medium, FC SBE RLU; fold change in SMAD-binding element relative light units. Statistical differences were calculated using two-way ANOVA followed by Sidak’s multiple comparisons test; *p* < 0.05 *, *p* < 0.01 **, and *p* < 0.001 ***.

The gene expression analysis revealed no straightforward mechanism for the TRPV4 increase in TGFβ signalling. However, as TRPV4 activation has a dependence on calcium, we investigated the effect of lowering the calcium concentration in the medium from 1800 µM (as in DMEM) to 72 µM. This prevented GSK101 enhancement of SBE-nLUCp induction (Figure 4A and Figure S13). In the complete absence of additional calcium within growth medium, TRPV4 activation caused a suppression rather than an enhancement of TGFβ signalling. As calmodulin can act as an intracellular calcium sensor and is known to interact with intracellular members of the TGFβ pathway, we hypothesised that TRPV4-mediated enhancement of TGFβ signalling involved calcium-bound calmodulin. To investigate this, we pre-incubated cells with calmodulin inhibitor KN93 prior to TGFβ3 and TRPV4 activation. The enhancement of TGFβ signalling after TRPV4 activation was found to be supressed by inhibition of calmodulin, indicating its role in transducing TRPV4-related activity (Figure 4B and Figure S13).

### 3.8. TRPV4 Activation Enhances TGFβ Signalling through JUN and SP1

**Figure 5:**
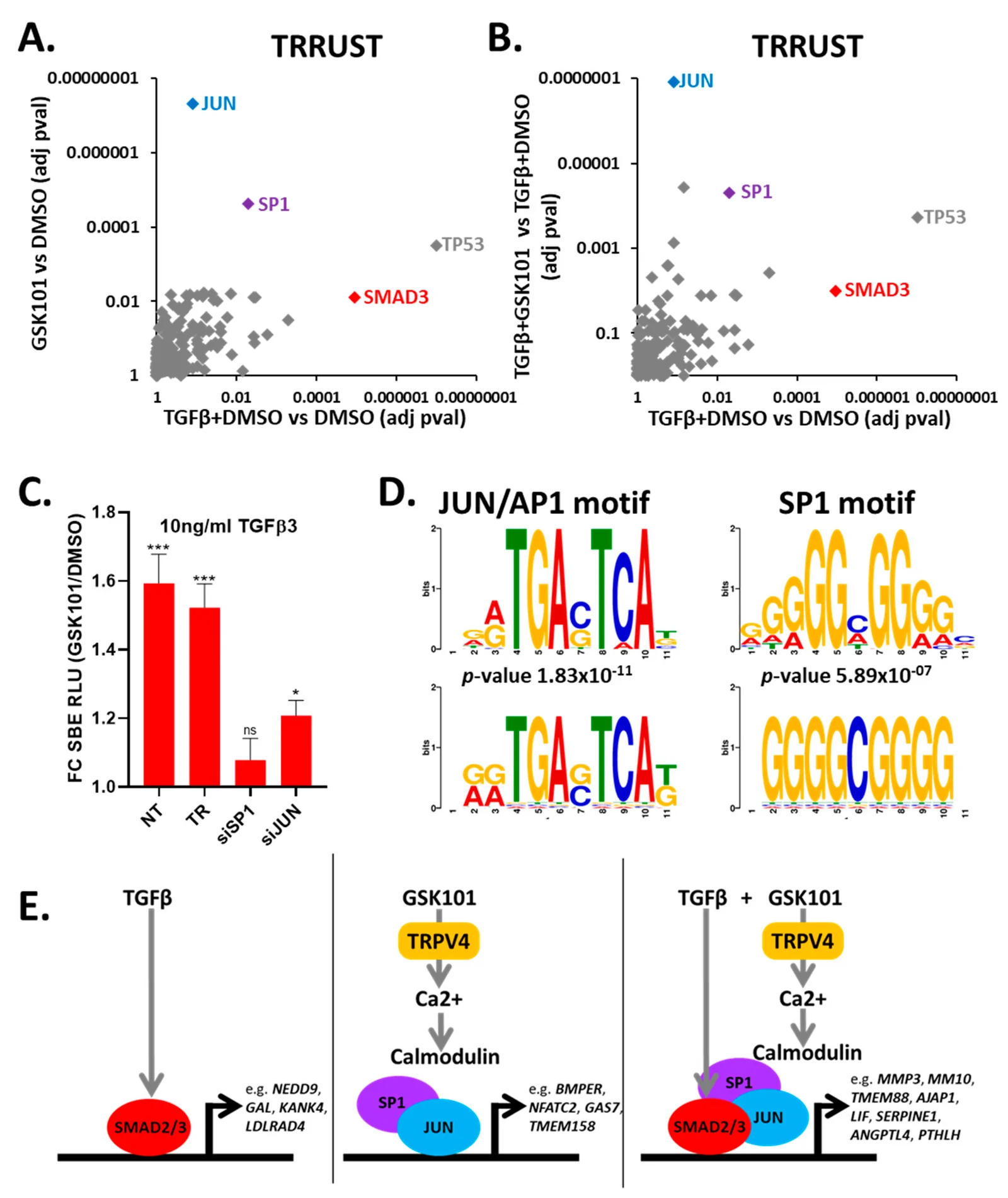
TRPV4 activation enhances TGFβ signalling through the JUN and SP1 transcription factors. (**A**,**B**) TRRUST analysis of genes significantly increased in RNAseq for each of the indicated experimental comparisons. (**C**) siRNA knockdown of JUN and SP1 prevents TRPV4 enhancement of TGFβ signalling. Data were combined from three independent experiments. GSK101 treatment was normalised to DMSO for each siRNA. Statistical differences were calculated using two-way ANOVA followed by Sidak’s multiple comparisons test; *p* < 0.05 * and *p* < 0.001 ***. FC SBE RLU; fold change in SMAD-binding element relative light units. (**D**) Sequence motif logos of JUN (MA0490.1; *p*-value 1.83 × 10^−11^) and SP1 (MA0079.3.1; *p*-value 5.89 × 10^−7^) within upregulated genes following TRPV4 activation created by TOMTOM from JASPAR2018_CORE_vertebrates_non-redundant database. (**E**) Schematic representation of possible mode of action of TGFβ and GSK101. In the presence of only TGFβ, SMAD2/3 causes transcriptional response of TGFβ target genes. In the presence of only GSK101, TRPV4 is activated, causing increased intracellular calcium, leading to activation of TRPV4 response genes. When TGFβ stimulation is followed by TRPV4 activation after 15 min, TGFβ activates SMAD3, and then TRPV4 activation causes increased calcium, enhancing the effect of TGFβ, through a mechanism involving SP1 and JUN, which are known SMAD3-binding partners. NT, no treatment; TR, transfection reagent control.

As TRPV4-mediated enhancement of TGFβ signalling occurred within 1 h (Figure 2F), we investigated the involvement of key regulatory transcription factors. The TRRUST transcription factor database was used to identify enrichment for transcription factors known to control the genes whose expression increased following TGFβ3 stimulation and TRPV4 activation. From these genes, we identified an enrichment of SMAD3 targets (Figure 5A,B, Table S5), a transcription factor mediating downstream TGFβ responses. SMAD3 targets were also enriched following TRPV4 activation, which is consistent with TRPV4 activation involving canonical TGFβ signalling (Table S5, Figure 5A,B). Genes whose expression increased following TRPV4 activation were most significantly enriched for known target genes of JUN and SP1 (Figure 5A,B). An enrichment of JUN targets was also detected using the ChEA database (Figure S14, Table S6). To investigate this, we separately knocked down JUN and SP1 using siRNA, which in both cases reduced TRPV4-mediated enhancement of TGFβ signalling (Figure 5C and Figure S14). G:profiler analysis of genes whose expression increased following TRPV4 activation identified a significant enrichment for the JUN- and SP1-binding motifs (Figure 5D). The expression of some members of the AP-1 transcription factor complex were also increased following TRPV4 activation (Figure S15). However, SP1 transcript expression was not increased following TRPV4 stimulation (Figure S15). Taken together, these data suggest TRPV4 activation enhances TGFβ signalling through a mechanism involving intracellular calcium/calmodulin and the transcription factors JUN and SP1 (Figure 5E).

## 4. Discussion

### 4.1. TRPV4 Activates Canonical TGFβ Signalling

Despite their shared involvement in a number of essential differentiation and phenotypic functions of chondrocytes, TRPV4 has not previously been considered to have any regulatory control of canonical TGFβ signalling. In other systems, some indirect links to TGFβ signalling through non-canonical pathways have been proposed. In cardiac fibroblasts, TRPV4 is required for differentiation to myofibroblasts [40] and evidence suggested that this is driven by Rho/Rho kinase activation downstream of TRPV4 [41]. TRPV4 regulation of EMT in mouse epidermal keratinocytes is through the non-canonical PI3K/AKT pathway [38] and in hippocampus, TRPV4 action was reported on the non-canonical P38-MAPK pathway [39]. Some target mechanisms of TRPV4 thus appear to be more effected in certain cells, suggesting a cell type-specific bias.

In this chondrocyte study, changes in gene transcription due to TRPV4 activation occurred much earlier than previously reported. Increased nuclear SMAD2 translocation and enhanced SBE-nLUCp was observed after only 15 and 45 min of pharmacological TRPV4 activation, respectively, suggesting a rapid and direct effect of TRPV4 activation on canonical TGFβ signalling. As TGFβ3 has been shown to drive a stronger chondrogenic response than TGFβ1 in bone marrow-derived mesenchymal stromal cells [42], we routinely used TGFβ3 in this study. However, our data with TGFβ1 indicates that modulation of TGFβ signalling is not TGFβ ligand specific. Bioinformatic analysis of the >1000 genes whose expression increased within 4 h of TRPV4 activation revealed an enrichment for known SMAD3 target genes, confirming that, in chondrocytes, TRPV4 activates the expression of a whole spectrum of canonical TGFβ targets.

### 4.2. Mechanism

As TRPV4 stimulates extracellular calcium influx and was able to increase SBE-nLUCp activity within 1 h of activation, we wanted to investigate whether TGFβ signalling enhancement is dependent upon extracellular calcium. Indeed, reduced external calcium (72 µM) prevented TRPV4 enhancement of TGFβ signalling. Calcium and calmodulin are both directly linked to TGFβ signalling; activation of TGFβ signals can result in Ca2+ influx [43], and calmodulin can directly interact with members of the TGFβ signalling pathway [44,45,46]. Surprisingly, at even lower calcium concentrations (<15 µM), the effect of TRPV4 activation was to inhibit TGFβ signalling. The reason for this is unclear but one possibility is that in the absence of external calcium, activation of TRPV4 results in internal calcium efflux, thus disrupting membrane potential and suppressing calcium/calmodulin-dependent pathways such as in the TGFβ signalling pathway [43,47]. This may involve blocking the nuclear–cytoplasmic shuttling of SMADs that would lead to inhibition of nuclear accumulation and failure to activate downstream target genes.

To identify specific transcription factors which may mediate the enhancement of TGFβ signalling by TRPV4 activation, we analysed our RNA-seq data using the TRRUST transcription factor network database of target interactions [48]. We analysed the 1991 genes that increased following TGFβ3 stimulation and identified that SMAD3 was a key transcription factor in the response to TGFβ3 as expected, thus validating the relevance of this approach to our chondrocyte system. Similar analysis of the 1448 genes upregulated following TRPV4 activation indicated that JUN (part of the AP-1 transcription factor complex) and SP1 were key mediators of the TRPV4 response. This was confirmed using siRNA to JUN and SP1, which separately reduced the response to TRPV4 activation. It is therefore likely that TRPV4 functions in a similar way to TRPV1, reported to cause calcium-dependent AP-1 transcription factor activation [49]. The involvement of the AP-1 transcription factor is consistent with its rapid activation following mechanical loading [50]. JUN and SP1 are also known SMAD co-factors [18,51], suggesting their interaction may enhance SMAD translocation and TGFβ-mediated gene transcription. There was only a modest enrichment for SP1 and no enrichment for JUN targets following TGFβ3 stimulation without TRPV4 activation, suggesting that TGFβ3 stimulation alone is not enough to activate SP1 and JUN, in this system. It was notable that one of the genes synergistically enhanced by TGFβ3 stimulation and TRPV4 activation is MMP3, which was previously shown to be regulated by JUN [52] and is responsive to loading in a rat post-traumatic model of OA [53]. As well as enrichment for TGFβ signalling, our bioinformatic analysis revealed enrichment for focal adhesion pathway genes. Focal adhesions are also important for TGFβ regulation [37] and TRPV4 is known to directly bind β1 integrin [54] a key component of cell–substrate focal adhesions, adding further complexity to the TRPV4–TGFβ interaction.

### 4.3. TRPV4 Modulation of TGFβ Signalling Is Highly Dependent upon Timing

TRPV4 activation using the potent and specific activator GSK101 [35] had a rapid, within 1 h, effect on the TGFβ transcriptional response. The sequence of TRPV4/TGFβ activation was also found to be critical. TRPV4 enhanced TGFβ signalling when activated after TGFβ3 stimulation and inhibited signalling when TRPV4 was activated before TGFβ3 stimulation. The underlying mechanisms that drive these differences require further investigation. Following TRPV4 activation we also observed decreased expression of *BAMBI* and increased expression of *FST* and *LTBP1*, which are all known to inhibit TGFβ responses. These apparent contradictions also require further investigation, although the increase in some TGFβ inhibitors may explain why the TRPV4-mediated increase in TGFβ response is greatest at 1 h and then diminishes with time, and possibly why the association between TRPV4 and canonical TGFβ signalling has not previously been reported. We also observed a modest increase in TGFβ signalling when endogenous TRPV4 expression was knocked down, revealing the presence of a low level of TRPV4 activity in the absence of external stimuli [2] and that TRPV4 may have an inhibitory effect on TGFβ signalling when assessed over longer periods of time. There is clearly a complex relationship between TGFβ and TRPV4 responses in chondrocytes and although their interaction is now established, the precise detailed mechanisms involved will require further investigation.

The TRPV4 inhibitor GSK219 [36] was able to completely prevent the enhancement of TGFβ signalling by TRPV4. GSK219 did not affect TGFβ signalling unless TRPV4 was activated, showing it to be highly selective. However, when GSK219 was added 15 min after TRPV4 activation (rather than 15 min before), it was ineffective. This is consistent with the rapid opening of the TRPV4 channel in response to activation [54], and further suggests that TRPV4 can activate a cascade of signals affecting the TGFβ signalling pathway, although sustained TRPV4 activation is not required for TGFβ signalling.

### 4.4. Downstream Implications of TRPV4–TGFβ Pathway Interaction

A large number of genes showed changes in expression following TRPV4 activation, including *MMP3*, *MMP10* and *SERPINE1* which are all involved in matrix turnover. This suggests that TRPV4 activation, for instance through mechanical loading, may play a role in ECM remodelling, for example in the joint following exercise or when loading becomes abnormal after injury or OA. TRPV4 may contribute to the altered TGFβ signalling that occurs in the OA affected joint [23], since we show that TRPV4 can increase TGFβ signalling and its expression has been shown to be increased in OA [12]. TRPV4 has been identified as a regulator of the chondrogenic phenotype via the enhancement of SOX9-driven COL2A1 expression [6]. The data presented here suggest that this may be through its effect on TGFβ signalling. Furthermore, patient-derived iPSCs with TRPV4 mutation have an altered response to TGFβ after chondrogenic differentiation [55].

## 5. Conclusions

It is established that TRPV4 activation increases intracellular calcium and plays an important role in cartilage development and maintenance. However, the mechanism by which it does so is not well established. Here we show that pharmacological TRPV4 activation can modulate the response to TGFβ stimulation in chondrocytes and establish the signalling pathway by which this occurs. We show that pharmacological TRPV4 activation enhances the effect of TGFβ in a calcium and calmodulin-dependent manner. The timing and the sequence of TRPV4 activation in relation to TGFβ stimulation are critical in determining the effect. Using RNAseq, we have identified TRPV4 responses and shown that TRPV4 enhancement of TGFβ signalling is JUN/SP1 dependent. Further research is required to establish whether these pathways are modulated in the same way in a more physiological environment, for example by mechanical loading in a three-dimensional tissue.

## Data Availability

The data presented in this study are openly available in the ArrayExpress database at EMBL-EBI under accession E-MTAB-10279.

## Supplementary Materials

The following are available online at https://www.mdpi.com/article/10.3390/cells10040726/s1, Figure S1: Validation of SBE-nLUCp reporter in TC28a2 cells; Figure S2: TRPV4 Western blot; Figure S3: Dose responses and raw data showing that activation of TRPV4 enhances TGFβ signalling; Figure S4: Activation of TRPV4 regulates TGFβ signalling in chondrocytes of several species; Figure S5: Timing of TRPV4 activation and inhibition is important for TGFβ signalling activity; Figure S6: Example genes from RNA-seq grouped based upon expression pattern; Figure S7: q-RT-PCR validation of example gene; Figure S8: TRPV4 modulation of the TGFβ signalling pathway; Figure S9: Gene expression changes within the Kegg ‘TGFβ signalling pathway’; Figure S10: TRPV4 modulation of the focal adhesion pathway; Figure S11: TRPV4 modulation of the PI3K/AKT signalling pathway; Figure S12: TRPV4 modulation of the MAPK signalling pathway; Figure S13: Reduction in extracellular calcium or calmodulin inhibition prevents GSK101 enhancement of TGFβ signalling; Figure S14: TRPV4 activation enhances TGFβ signalling through the JUN and SP1 transcription factors; Figure S15: Expression changes of genes encoding AP-1 complex and SP1; Table S1: Primer sequences for qRT-PCR; Table S2: Read count and differential gene expression analysis for indicated experimental comparison; Table S3: Differentially expressed genes for indicated experimental comparison; Table S4: Kegg pathways that are significantly enriched within upregulated genes for indicated experimental comparison; Table S5: TRRUST transcription factor analysis of upregulated genes for indicated experimental comparison; Table S6: ChEA transcription factor analysis of upregulated genes for indicated experimental comparison.
